# Variations in the *WDR36* gene in German patients with normal tension glaucoma

**Published:** 2007-05-16

**Authors:** Nicole Weisschuh, Christiane Wolf, Bernd Wissinger, Eugen Gramer

**Affiliations:** 1 Molecular Genetics Laboratory, University Eye Hospital, Tuebingen, Germany; 2 University Eye Hospital, Wuerzburg, Germany

## Abstract

**Purpose:**

To determine the prevalence of *WDR36* sequence variants in a cohort of German patients with normal tension glaucoma.

**Methods:**

All of the 23 coding exons and flanking introns of the *WDR36* gene were amplified by PCR from genomic DNA and subjected to denaturing high performance liquid chromatography. Samples with aberrant profiles were sequenced. In addition, restriction fragment length polymorphism analysis was performed in order to identify previously described nucleotide changes.

**Results:**

This study identified 11 nucleotide changes in the *WDR36* gene that lead to predicted amino acid substitutions. Previously reported disease-causing mutations were found in 4% of patients (4/112) whereas sequence variants previously classified as disease-susceptibility mutations were found in 5% of patients (6/112). One nonsynonymous nucleotide change that has not been reported before was found in one patient. Mutation screening also identified several exonic and intronic polymorphisms.

**Conclusions:**

The findings in the current study indicate that *WDR36* gene variants may be only rare causes of normal tension glaucoma in the German population.

## Introduction

The term glaucoma describes a heterogeneous group of optic neuropathies that lead to optic nerve atrophy and permanent loss of vision, affecting over 67 million subjects worldwide [1]. Since glaucoma rates rise exponentially with age, more glaucoma cases are expected in the future as the western population gets older [2]. The disease is insidious. Affected patients frequently have no symptoms, especially in the initial stages. When detected early, most cases can be successfully treated with medications, laser treatment, or surgery.

The hereditary forms of glaucoma are genetically heterogeneous. Primary open angle glaucoma (POAG) of adult onset is the most common form of glaucoma, representing approximately half of all cases. Normal tension glaucoma (NTG) is an important subtype of POAG, accounting for approximately 20-50% of all POAG cases. Patients with NTG show intraocular pressures that are within the statistical normal range of the population (10-20 mmHg).

Despite the rather high prevalence and heritable nature of POAG, identification of causative genes has proven to be difficult. Though at least 20 loci have been linked to POAG [3], only two disease-causing genes for POAG have been identified until lately. A number of studies confirmed that about 4% of POAG cases show mutations in the *MYOC* gene [4]. Although mutations in the optineurin gene (*OPTN*) were initially reported in 16.7% of families with hereditary POAG [5], several other studies have indicated that *OPTN* sequence variants are only a rare cause of POAG or NTG [6-8]. Recently, a new locus for POAG (*GLC1G*) and the disease-causing gene (*WDR36*) have been identified by Monemi and coworkers [9]. In their study, mutation analysis of *WDR36* in 130 unrelated affected individuals revealed a total of 25 allelic variants. The function of WDR36 and its role in the normal ocular physiology and glaucoma is still unclear, but WDR36 has been identified as being involved in T cell activation [10].

The purpose of this study was to determine the prevalence of *WDR36* sequence variants in a cohort of unrelated German patients with NTG.

## Methods

### Screening and selection of patients

The study population comprised a cohort of 112 unrelated white patients with NTG that included 67 women and 45 men. Their age range was 14-84 years with a mean age of 62.3±15.3. All study patients were part of a group of 289 patients that had been previously clinically investigated at the University Eye Hospital in Wuerzburg [11]. Selected from this group for molecular genetic analysis were 112 patients who had been followed long-term.This was to ensure diagnosis of NTG with a maximum of certainty. Patients have been screened for *MYOC* and *OPTN* mutations in a previous study [12]. Glaucoma was defined by the following strict criteria: (1) the presence of typical glaucomatous optic neuropathy with compatible visual field loss (according to Aulhorn classification) [13]; (2) open drainage angles on gonioscopy; and (3) absence of a secondary cause for glaucomatous optic neuropathy, such as a previously elevated intraocular pressure (IOP) after trauma, a period of steroid administration, or uveitis. Patients also did not have evidence of high myopia or congenital ocular abnormality, and had no other cause than glaucoma for disk changes and visual field loss. A neurological examination was performed in order to exclude an intracerebral expansion. Sonography was used to rule out aortic stenosis. Patients with untreated NTG had IOP measurements that were consistently 21 mmHg or lower on diurnal testing and during follow-up (IOP readings were correlated with corneal thickness). Disk size and parameters were evaluated by means of confocal examination (Heidelberg Retina Tomograph). Control DNA samples were obtained from 50 unrelated subjects of German descent who did not have a family history of glaucoma. Control individuals were age matched (mean age 60.5±18.7) and were collected from the same geographic region as the probands. A diagnosis of glaucoma was ruled out based on IOP measurements and ophthalmoscopy of the optic disk. Written informed consent was obtained from all participants. This study was approved by the ethics committees of the University Hospital Tuebingen and the University Hospital Wuerzburg. It was performed in accordance with the Helsinki Declaration.

### Detection of nucleotide variants by denaturing high performance liquid chromatography

Patient DNA was extracted from peripheral blood lymphocytes using a standard salting-out procedure. Individual exons of the *WDR36* gene were amplified by polymerase chain reaction (PCR) using appropriate amplification protocols. Primer pairs used for amplification are listed in Table 1. All amplicons containing the coding exons and neighboring intronic regions of *WDR36* were screened for nucleotide changes with the WAVE denaturing high-performance liquid chromatography system (Transgenomic, Inc., Elancourt, France). The PCR products to be tested were mixed with an equimolar amount of a known unmutated PCR product to form heteroduplexes. Temperatures used for analysis are given in Table 2. The resulting DHPLC trace profiles were examined with the Navigator^TM^ software (Transgenomic, Inc.). The chromatographs were compared with the profile of wild-type DNA fragments. Samples with aberrant profiles were sequenced.

**Table 1:** Primer pairs used for amplification of *WDR36*.

**Exon**	**Forward (5'-3')**	**Reverse (5'-3')**
1	AATCGTTTCCATCTCCAAGG	AGGACTGCAAGTGCCAAATC
2	TCTTATGAAGGACAGCATAGCAA	CACTGTGATTCCTCCCAAGG
3	GGGAAGGACAAGGTGATTTC	TTCCAGAGTGTGCTGGGTAA
4	GAGGGAGCAGATGAACATGC	CAGGCAAAATCTCTGGCATA
5	CATTTACAAGTTGCCTCTCATTT	CCTCTGATACAGGGGACCAA
6	TTCTTAATGAGGTGGCATAGCA	TTGGCAAAGGCATTATTACTTG
7	TGCTGAGTTTTTCTGCCATC	TTTGCCTTTTACTCCAGTATTCA
8	TGGATTAAAAGGGAAGAGAGAAGA	TCATCTTCTAGGTTGAAAGCTGAT
9	AATACCCACTCCCTCCCTTG	ACCCAGACTTCTAAGTCACTCAA
10	TGCAATCTGGTTTTCCCTTT	GCAACTTTGATGCTAGGAATCTT
11	CAGTGGTAATAACATCTTTGTTTTGT	ACAGAAGAGCAAAGCCTGATG
12	TGATTTAGCCGTTCCACAATG	CAATATTATGATGAGAAACCTTG
13	TGAAGCAATTCATTTATTGTTCTTTT	CCAGCACTTAAAGATTATAC
14	TTGAATGAAGGAATCACTGTGTG	TGGTAAATTGTGACTTTATGAC
15	GGCTTAATTTCCCCCAAAGT	ATCGCATCAACTCCCTGAAA
16	AGGCAGCCTGAATGTTAGTCA	AAGGTTTAGGCATCTCGCTTC
17	ACTTTTAACATGTTAATTATTTCTG	GTATCTGTGTTAACTTGTGAC
18	TGCTCAGCATATTCGCACTC	CCCATCACTAGACCCAAAACA
19	CCATTGAAGAAGGTGTTTTGG	TTGCTCTTCTAATGCCCTCAA
20	TATCGGCAAGGGTGCTAGAG	AATTCAAGTATCACCCAAAAATG
21	TTCTGCACTTCTTTACCAAGACC	TGAGAACGCTGATATTTCCTTC
22	TGTTGCAAGGCATTAGATTTTC	CTGCCCCAGAAGAAGACAAG
23	GGTGGAGAGGAGTGATGACC	GCTCAAATTCCTGGCTTCAA

Individual exons of the *WDR36* gene were amplified by polymerase chain reaction (PCR) using appropriate amplification protocols.

**Table 2:** Amplicon sizes and optimum temperatures for DHPLC analysis of all individual *WDR36* exons.

**Exon**	**Amplicon size (bp)**	**Temperatures (°C)**
1	724	62.1/63.0/63.6
2	500	53.8
3	414	57.1
4	402	53.8/55.1
5	469	53.5/54.5/56.5
6	465	54.0/55.0
7	396	55.5/55.9
8	361	57.8/58.3
9	392	57.0/57.4
10	362	56.5/57.0
11	450	54.5/55.5/56.0
12	531	55.9/56.5/57.2
13	450	55.5/56.5
14	376	55.4/56.5/57.5
15	400	57.4/58.7
16	534	56.1/57.3
17	282	55.2/57.6
18	695	54.1/56.3
19	438	53.9/54.9
20	485	56.0
21	301	54.2/55.1
22	516	58.0
23	643	54.3/54.9

Temperatures used for denaturing high performance liquid chromatography. In the table, bp indicates base pairs.

### Mutation detection by direct sequencing

PCR fragments were purified by ExoSAP-IT treatment (USB, Cleveland, OH), sequenced using Big Dye Termination chemistry (Applied Biosystems, Weiterstadt, Germany) and products separated on a DNA capillary sequencer (ABI 3100 Genetic Analyzer).

### Detection of nucleotide variants by restriction fragment length polymorphism analysis

A 361 bp PCR product encompassing exon 8 of the *WDR36* gene was digested with 1 U *Hpy*CH4III restriction enzyme (NEB, Beverly, MA). The N355S missense change resulted in the gain of an *Hpy*CH4III restriction site that, upon cleavage of the amplicon, produced two DNA fragments of 251 bp and 110 bp.

A 450 bp PCR product encompassing exon 11 of the *WDR36* gene was digested with 1 U *Tse*I restriction enzyme (NEB). The A449T missense change resulted in the loss of a *TseI* restriction site. Wild-type sequences were cleaved into two fragments of 229 bp and 221 bp.

A 450 bp PCR product encompassing exon 13 of the *WDR36* gene was digested with 1 U *Taq*I restriction enzyme (NEB). The R529Q missense change resulted in the loss of a *Taq*I restriction site. Wild-type sequences were cleaved into two fragments of 232 bp and 218 bp.

A 282 bp PCR product encompassing exon 17 of the *WDR36* gene was digested with 1 U *Bgl*I restriction enzyme (NEB). The D658G missense change resulted in the gain of a *Bgl*I restriction site that, upon cleavage of the amplicon, produced two DNA fragments of 233 bp and 49 bp. Restriction digests were analyzed on a 4% agarose gel.

## Results

**Figure 1:**
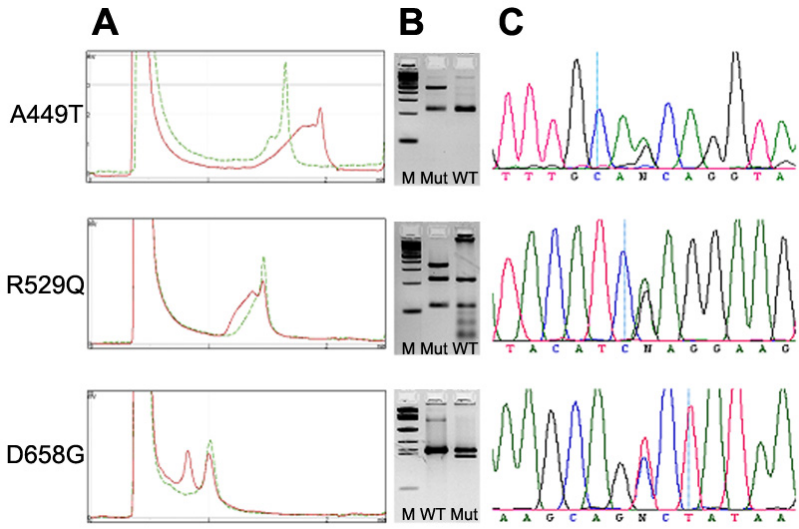
Comparison of three mutation detection systems used in this study. **A**: Denaturing high performance liquid chromatography profiles of mutant (red) and wild-type (green) alleles of three *WDR36* missense mutations. **B**: RFLP analysis. The following abbreviations were used: 100 bp ladder (M), restriction digest of mutant PCR product (Mut), and restriction digest of control PCR product (WT). **C**: DNA sequence analysis.

We screened the complete coding sequences plus flanking intron/UTR sequences of the *WDR36* gene by denaturing high performance liquid chromatography (DHPLC) and subsequent sequencing in 112 patients with NTG. In addition, we performed RFLP analysis for four known sequence alterations that were judged in a previous study to be disease-causing mutations [9]. Of those four, we were able to identify three by RFLP analysis as well as with DHPLC and subsequent sequencing, namely A449T (found in two subjects), R529Q (found in one subject), and D658G (found in one subject), respectively (Figure 1 and Table 3). We were not able to detect any of these changes in 50 controls using DHPLC and RFLP analysis.

**Table 3:** Frequencies, localization, and putative functional effect of identified *WDR36* sequence variants.

**Nucleotide change**	**Predicted protein change**	**Prediction of functional effect**	**Observed variants/ no. of patients**	**Observed variants/ no. of controls**
Non-synonymous changes				
c.l345G>A	A449T	benign	2,112	0/50
c.1586G A	R529Q	possibly damaging	1/112	0 50
e. 1973A G	D65SG	probably damaaina	1 112	0/50
c.91C>A	P31T	unknown	1 112	0/50
c.99C>G	D33E	unknown	2/112	0 50
c.4S8C T	A163V	benign	2 112	0 50
c.635 636delinsCC	H212P	probably damaging	2/112	0 50
Synonymous changes				
c.591G>A	Q197Q		2/112	ND
cl692A>G	K564K		1/112	ND ND
c.2142C>G	V714V		17/112	ND ND
c.2181A >T	V727V		36/112	(SNP: rsl3186912)
Intronic changes				
c.460-113G>A			15/112	ND
c.460-159G A			9/112	ND
c.710+30C>T			50/112	(SXP: rsl0038177)
c.898+105A G			2/112	ND
c.1074+56T>C			1/112	ND
c. 1075-1 15G A			1/112	ND
c.l348+30G>T			1/112	ND
c.1609-89G A			8/112	ND
c.2518-60G>C			15 112	(SNP: rs2290680)
c.*83A>T			1 112	ND
c.*93A>C			5/112	ND

Sequence variants identified in the *WDR36* gene. The "Nucleotide change" column is based on GenBank accession number NM_139281 and the "Prediction of functional effect" column is according to PolyPhen. In the table, ND refers to not determined.

DHPLC analysis and subsequent sequencing also identified four additional nonsynonymous nucleotide changes, namely P31T, D33E, A163V, and H212P which were not present in 50 controls. The latter three have already been described [9,14], whereas this is the first report of P31T.

DHPLC screening and subsequent sequencing also revealed several synonymous polymorphisms (Q197Q, K564K, V714V, V727V) as well as 11 intronic alterations (Table 3). Neither a deletion/insertion nor a sequence alteration close to splice donor/acceptor sites change was found.

Four patients harbored a potentially causative mutation in the *OPTN* gene or *MYOC* gene as was found in a previous study [12]. These patients did not show sequence variants in the *WDR36* gene.

## Discussion

This study describes mutational screening in the *WDR36* gene in a large cohort of German patients with the NTG subtype of POAG. The primary aim of our study was to evaluate the prevalence of previously described mutations classified as disease causing [9]. Therefore, two different mutation-screening techniques (DHPLC and RFLP) were used to make sure that all four mutations (N355S, A449T, R529Q, and D658G) were detected.

**Figure 2:**
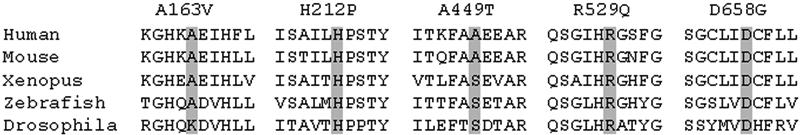
Amino acid alignment and evolutionary conservation of non-synonymous changes identified in this study. Partial amino acid sequences of WDR36 protein orthologs from five different species were aligned to show possible conservation. Grey boxes highlight affected residues. P31T and D33E are not shown as the very NH_2_-terminal part of the human WDR36 protein was not conserved.

None of our patients had the N355S mutation described by Monemi and coworkers [9]. Instead, we identified one patient who harbored the R529Q mutation that has already been described in two independent studies [9,14]. According to the prediction program PolyPhen, this particular change lies in a highly conserved region and possibly has a damaging effect (Table 3 and Figure 2). In addition, we identified two patients with NTG who carried the A449T sequence variant. This mutation is not part of a known protein motif. It was not conserved and is therefore benign according to the PolyPhen prediction. The D658G mutation noted in 3 out of 272 sporadic cases in the Monemi study was observed in one patient in the NTG group. In the former study this change was shown to be not present in 238 controls, whereas a case-control study on this particular variant in Australian patients demonstrated an equal distribution in both patients and controls [15]. Although this mutation leads to a conservative exchange of two polar and acidic residues, PolyPhen predicts this change to be probably damaging due to the high conservation between species (Figure 2).

In addition to the aforedescribed disease-causing mutations, we were able to identify sequence variants previously classified as disease-susceptibility mutations, namely D33E, A163V, and H212P [9,14]. We also noted one nonsynonymous nucleotide change (P31E) that has not been reported before. It was found in one patient in the NTG group and was not present in the control population. As the proline residue is not conserved between species (data not shown) and is not located in any known protein motif of WDR36, this change is probably benign.

Although our patient and control cohort numbers were too small for a statistical analysis to determine whether the sequence variants we identified might be related to the disease, our results indicate that sequence variants of the *WDR36* gene already described to be disease-causing are only rare causes of unrelated NTG in the German population.

Apart from the findings of our study concerning *WDR36* sequence variants, we observed that the results of the DHPLC screening in those exons that harbored already described mutations were consistent with the results of RFLP analysis (Figure 1). In addition, we identified several exonic and intronic polymorphisms by using DHPLC and subsequent sequencing. Therefore we reason that screening of the *WDR36* gene with DHPLC gives rather reliable results compared with the hands-on time. Direct sequencing still represents the gold standard in mutation screening, and other screening mechanisms, such as DHPLC, are not as sensitive. Nevertheless, we are convinced that our screening procedure using DHPLC and RFLP gave comparable good detection rates. From our study it seems likely that DHPLC gives false positive results rather than false negative ones. Yet, we have to consider that we may have missed some nucleotide changes that could be associated with the NTG phenotype in our patients. In addition, as we only screened for mutations in exonic and flanking intron sequences, it is possible that a proportion of our patients carry mutations in the promoter region or in other regulatory sequences of *WDR36*. Screening with either DHPLC or direct sequencing does not reveal gross insertions or deletions that may be responsible for the phenotype.

Our study indicates that *WDR36* sequence variants are only rare causes of unrelated NTG in the German population. In a previous study [12], we showed this also holds true for the other two POAG-associated genes *MYOC* and *OPTN*. As early diagnosis is critical for successful treatment of glaucoma, defining the genetic basis of hereditary forms of glaucoma is an important step towards a presymptomatic screening of people at risk. However, a routine genetic testing for the three known POAG-associated genes seems to be of limited value due to the low prevalence of disease-causing mutations.

A considerable number of studies dealing with the genetics of glaucoma have shown that only a small proportion of glaucoma cases are due to monogenic forms. The vast majority of cases are inherited as a complex trait. Therefore, genome-wide association studies might be a better strategy to use in defining genetic profiles for those at risk for developing glaucoma.
